# Metabolic engineering of *Methylobacterium extorquens* AM1 for the production of butadiene precursor

**DOI:** 10.1186/s12934-018-1042-4

**Published:** 2018-12-20

**Authors:** Jing Yang, Chang-Tai Zhang, Xiao-Jie Yuan, Min Zhang, Xu-Hua Mo, Ling-Ling Tan, Li-Ping Zhu, Wen-Jing Chen, Ming-Dong Yao, Bo Hu, Song Yang

**Affiliations:** 1 School of Life Sciences, Shandong Province Key Laboratory of Applied Mycology, and Qingdao International Center on Microbes Utilizing Biogas, Qingdao Agricultural University, Qingdao, Shandong China; 2 Key Laboratory of Systems Bioengineering, Ministry of Education, Tianjin University, Tianjin, China; 3 Industrial Product Division, Intrexon Corporation, South San Francisco, CA 94080 USA; 4 Marine Science and Engineering College, Qingdao Agricultural University, Qingdao, Shandong China

**Keywords:** *Methylobacterium extorquens*, Butadiene, Crotyl diphosphate, High throughput screening, In vitro reaction, Pathway engineering

## Abstract

**Background:**

Butadiene is a platform chemical used as an industrial feedstock for the manufacture of automobile tires, synthetic resins, latex and engineering plastics. Currently, butadiene is predominantly synthesized as a byproduct of ethylene production from non-renewable petroleum resources. Although the idea of biological synthesis of butadiene from sugars has been discussed in the literature, success for that goal has so far not been reported. As a model system for methanol assimilation, *Methylobacterium extorquens* AM1 can produce several unique metabolic intermediates for the production of value-added chemicals, including crotonyl-CoA as a potential precursor for butadiene synthesis.

**Results:**

In this work, we focused on constructing a metabolic pathway to convert crotonyl-CoA into crotyl diphosphate, a direct precursor of butadiene. The engineered pathway consists of three identified enzymes, a hydroxyethylthiazole kinase (THK) from *Escherichia coli*, an isopentenyl phosphate kinase (IPK) from *Methanothermobacter thermautotrophicus* and an aldehyde/alcohol dehydrogenase (ADHE2) from *Clostridium acetobutylicum*. The *K*_*m*_ and *k*_*cat*_ of THK, IPK and ADHE2 were determined as 8.35 mM and 1.24 s^−1^, 1.28 mM and 153.14 s^−1^, and 2.34 mM and 1.15 s^−1^ towards crotonol, crotyl monophosphate and crotonyl-CoA, respectively. Then, the activity of one of rate-limiting enzymes, THK, was optimized by random mutagenesis coupled with a developed high-throughput screening colorimetric assay. The resulting variant (THK^M82V^) isolated from over 3000 colonies showed 8.6-fold higher activity than wild-type, which helped increase the titer of crotyl diphosphate to 0.76 mM, corresponding to a 7.6% conversion from crotonol in the one-pot in vitro reaction. Overexpression of native ADHE2, IPK with THK^M82V^ under a strong promoter *mxaF* in *M. extorquens* AM1 did not produce crotyl diphosphate from crotonyl-CoA, but the engineered strain did generate 0.60 μg/mL of intracellular crotyl diphosphate from exogenously supplied crotonol at mid-exponential phase.

**Conclusions:**

These results represent the first step in producing a butadiene precursor in recombinant *M. extorquens* AM1. It not only demonstrates the feasibility of converting crotonol to key intermediates for butadiene biosynthesis, it also suggests future directions for improving catalytic efficiency of aldehyde/alcohol dehydrogenase to produce butadiene precursor from methanol.

**Electronic supplementary material:**

The online version of this article (10.1186/s12934-018-1042-4) contains supplementary material, which is available to authorized users.

## Background

Butadiene is the simplest conjugated diene and a major commodity of the petrochemical industry, used for the manufacture of automobile tires, synthetic resins, latex and plastics [1]. It is one of the most widely-used chemicals in the world with over ten million tons produced per year [2]. Currently, butadiene is predominantly synthesized from non-renewable petroleum as a byproduct of ethylene production [3]. Butadiene can also be produced by the dehydrogenation of butane or butene, but this process has some drawbacks including large consumption of steam and harsh operation conditions [4]. In contrast with traditional approaches, microbial synthesis of butadiene from renewable sources such as lignocellulose, biogas and carbon dioxide represents an environmentally friendly approach requiring lower investment [5, 6]. So far, there is no successful example of butadiene synthesis using biological platforms, despite the fact that three different pathways for butadiene production have been proposed, starting with crotonyl-CoA, erythrose-4-phosphate and malonyl-CoA respectively [2].

**Fig. 1:**
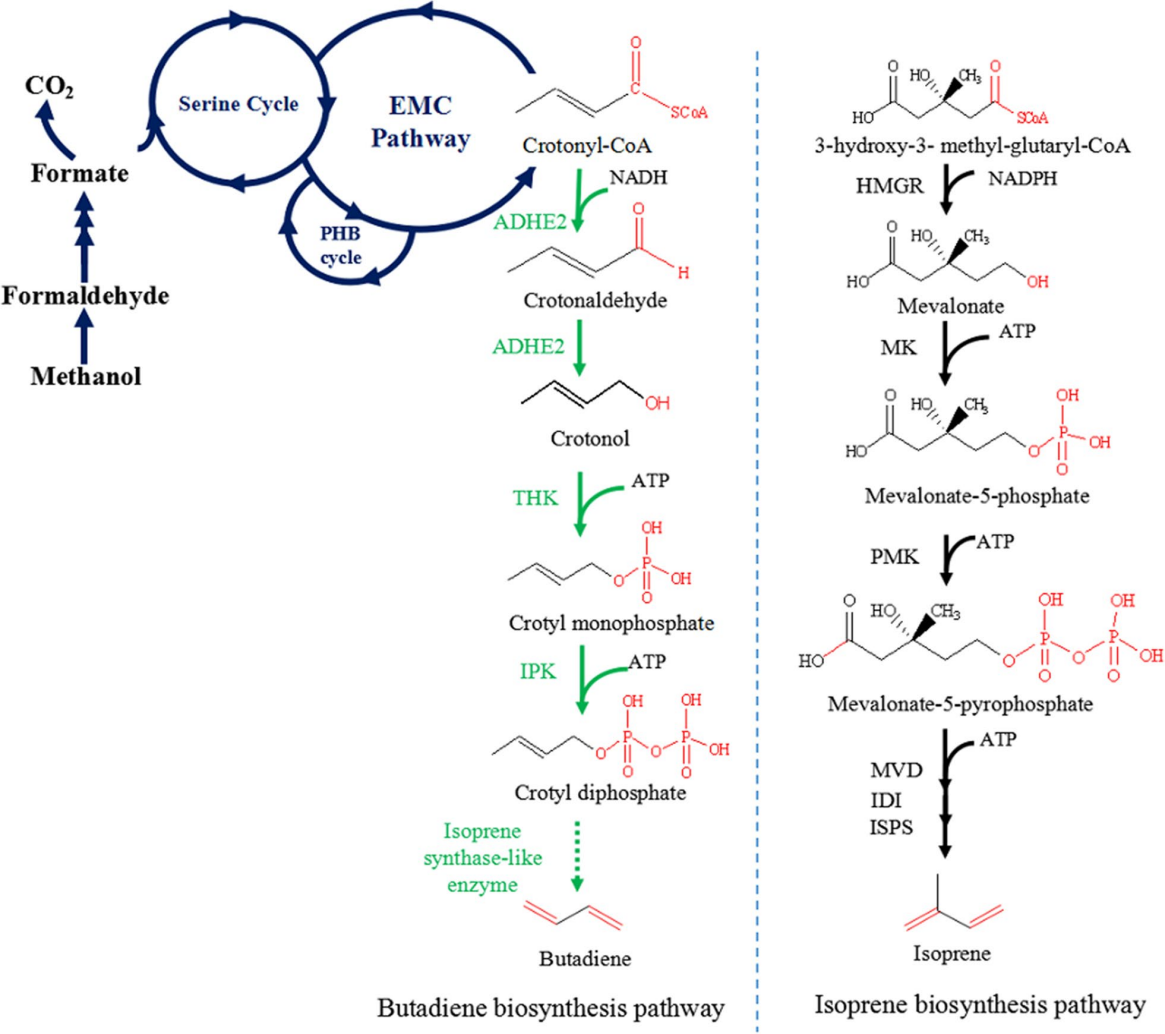
Proposed butadiene biosynthesis pathway in *M. extorquens* AM1. Blue lines represent methylotrophic pathways used for methanol assimilation. Green solid lines represent the partial butadiene pathway studied in this work. Black lines represent the isoprene biosynthesis pathway as a comparison. HMGR: 3-hydroxy-3-methyl-glutaryl-CoA reductase; MK: mevalonate kinase; PMK: phosphomevalonate kinase; MVD: mevalonate-5-diphosphate decarboxylase; IDI: isopentenyl pyrophosphate isomerase; ISPS: isoprene synthase; ADHE2: aldehyde/alcohol dehydrogenase; THK: hydroxyethylthiazole kinase; IPK: isopentenyl phosphate kinase

*Methylobacterium extorquens* AM1, a facultative methylotrophic α-proteobacterium, is capable of utilizing methanol as the sole carbon and energy source [7]. Methanol is known as an important C1 feedstock, which can be generated from synthesis gas (a mixture of CO and H_2_) or from biogas with relatively cheap cost [8]. The methylotrophic metabolism in *M. extorquens* AM1 involves three interlocked cycles: the serine cycle, the ethylmalonyl-CoA pathway (EMC pathway) and the poly-3-hydroxybutyrate (PHB) cycle (Fig. 1) [9]. Notably, crotonyl-CoA is an important intermediate in the EMC pathway [10–12], providing a stable supplement as a precursor for producing value-added chemicals [13, 14]. Hu et al. constructed a heterologous pathway for converting crotonyl-CoA into 1-butanol in *M. extorquens* AM1 as an early example of such metabolic engineering [15, 16]. Another example is increased yield of crotonic acid from crotonyl-CoA by expression of CoA-thioesterase from *Escherichia coli* [17]. Moreover, among the three proposed pathways, production of butadiene from crotonyl-CoA requires the fewest steps, with five sequential reactions: (i) reduction of crotonyl-CoA to crotonaldehyde, (ii) reduction of crotonaldehyde to crotonol, (iii) phosphorylation of crotonol to crotyl monophosphate, (iv) phosphorylation of crotyl monophosphate to crotyl diphosphate and (v) conversion of crotyl diphosphate to butadiene by an isoprene synthase-like enzyme ([2, 18]; Fig. 1). Therefore, the rationale of constructing a butadiene biosynthetic pathway from crotonyl-CoA in *M. extorquens* AM1 is well established.

One of the major challenges in this work is the lack of reports on enzymes catalyzing the biochemical reactions from crotonyl-CoA to butadiene. Herein, an in vitro one-pot strategy was applied for sequentially converting crotonol into crotyl diphosphate. The method simply mixes either purified enzymes or crude lysates in a single reaction vessel, presenting the advantage of easy process control and optimization [19, 20]. More importantly, this strategy is able to efficiently identify promiscuous enzymes contributing to butadiene synthesis and thus provides an important basis for further engineering in *M. extorquens* AM1.

In this study, we identified three enzymes that catalyze the conversion of crotonyl-CoA to crotonol and the subsequent phosphorylation into crotyl monophosphate and crotyl diphosphate. Next, we developed a high-throughput screening method to isolate a kinase variant for significantly improving the catalytic efficiency towards crotonol, which led to increased production of crotyl diphosphate in the one-pot reaction system. Furthermore, we heterologously overexpressed three genes in *M. extorquens* AM1 and the engineered strain was able to produce crotyl diphosphate with the feeding of crotonol. This research sheds light on the production of butadiene from methanol in *M. extorquens* in the future.

## Methods

### Culture medium and condition

Cultures of *E. coli* strain Top10 and *E. coli* strain BL21-Z were grown at 37 °C in Luria–Bertani (LB) medium. *M. extorquens* AM1 and its recombinant strains were routinely cultured in a minimal medium as described previously [21]. Briefly, the strains were first inoculated in tubes and pre-cultivated to mid-exponential phase at 30 °C, and then 0.5 mL of a sub-culture was transferred into 50 mL of minimal medium in 250 mL flasks and grown on rotary shaker at 200 rpm. Substrates and antibiotics were supplied at the following concentrations: succinate (15 mM), methanol (125 mM), 20 μg/mL tetracycline (Tet) and 50 μg/mL ampicillin. MC minimal medium was adapted from the previous description to cultivate *M. extorquens* AM1 in 96-well plates [22]. All chemicals were purchased from Sigma-Aldrich (St. Louis, MO, USA) unless otherwise specified. Crotyl monophosphate and crotyl diphosphate were synthesized by KareBay Biochem, Inc (Ningbo, Jiangsu Province, China). Milli-Q (Billerica, MA, USA) was used for preparing all the media, buffers, standards, and sample solutions.

### Plasmids and strain construction

The *erg12* gene encoding mevalonate kinase from *Saccharomyces cerevisiae* (GenBank accession: BK006946.2), *thik* gene encoding thiamine kinase from *Salmonella enterica* (GenBank accession: AMG28242.1), *MTH_47* encoding isopentenyl phosphate kinase (IPK) from *Methanothermobacter thermautotrophicus* (GenBank accession: AAB84554.1), *far* gene encoding fatty acyl-CoA reductase (FAR) from *Hahella chejuensis* and *Marinobacter manganoxydans* (GenBank accession: ABC31684.1, WP_008171430.1), and *adhe*2 gene encoding aldehyde/alcohol dehydrogenase (ADHE2) from *Clostridium acetobutylicum* (GenBank: AF321779.1) were synthesized into the vector pUC57 (GenScript, Nanjing, China) with codon usage optimized for expression in *M. extorquens* AM1. The *thiM* gene encoding hydroxyethylthiazole kinase (THK) from *E. coli* K-12 (GenBank accession: AVI56602.1), and *gck* gene encoding glycerate kinase (GCK) from *M. extorquens* AM1 were amplified from the genomic DNA [23]. The genes were amplified using the corresponding primers listed in Additional file 1: Table S1. Resulting PCR products of *erg12*, *thik*, *MTH_47*, *thiM*, and *gck* genes were digested with *Bam*HI and *Sac*I. The PCR products of *far* and *adhe2* were digested with *Hin*dIII and *Bam*HI. The fragments of genes were assembled into the same restriction sites of pCM80 [24] and pET.32M.3C. All the plasmids were transformed into *M. extorquens* AM1 by electroporation as described before [25] or into *E. coli* BL21-Z, respectively (Table 1).

**Table 1:** Strains and plasmids used in this study

Strains or plasmids	Description	Source or references
Strains		
*E. coli* BL21-Z	F^−^*ompT hsd*S_B_(r_B__m_B_^−^)*gal dcm*	A gift from Dr. Yu-Long Zhao at the Tianjin Medical University
*M. extorquens* AM1	Wild-type, pink color, rifamycin-resistant strain	[21]
YCB0	*M. extorquens* AM1/pCM80	This study
YJM	*M. extorquens* AM1/pCM80-*thiM*	This study
YJK	*M. extorquens* AM1/pCM80-*thik*	This study
YJG	*M. extorquens* AM1/pCM80-*erg12*	This study
YCB1	*M. extorquens* AM1/pCB1	This study
YCB3	*M. extorquens* AM1/pCB3	This study
YCB4	*M. extorquens* AM1/pCB4	This study
Plasmids		
pCM80	*M. extorquens* expression vector, *mxaF* promoter; Tc^R^	[24]
pET.32M.3C	Expression vector, T7 promoter, Amp^r^	Lab storage
pCM80-*thiM*	*thiM* from *E. coli* K-12 inserted into pCM80	This study
pCM80-*thik*	*thik* from *S. enterica* inserted into pCM80	This study
pCM80-*erg12*	*erg12* from *S. cerevisiae* inserted into pCM80	This study
pCM80-*far*	*far* from *Hahella chejuensis* inserted into pCM80	This study
pET.32M.3C-*thiM*	*thim* from *E. coli* K-12 inserted into pET.32M.3C	This study
pET.32M.3C-*thik*	*thik* from *S. enterica* inserted into pET.32M.3C	This study
pET.32M.3C-*erg12*	*erg12* from *S. cerevisiae* inserted into pET.32M.3C	This study
pET.32M.3C-*ipk*	*ipk* from *M. thermautotrophicus* inserted into pET.32M.3C	This study
pET.32M.3C-*gck*	*gck* from *M. extorquens* AM1 inserted into pET.32M.3C	This study
pET.32M.3C-*far*	*far* from *Hahella chejuensis* inserted into pET.32M.3C	This study
pCB1	pCM80 (PmxaF::*thim*::MTH_47)	This study
pCB3	pCM80 (PmxaF::*adhE2*::*thim*::MTH_47)	This study
pCB4	pCM80 (PmxaF::*adhE2*)	This study

PCR was performed using PrimeSTAR HS, and error-prone PCR was performed using Taq™ (Tankala, Dalian, China). The restriction enzymes were purchased from Fisher Scientific (Pittsburgh, PA, USA). T4 ligase was purchased from Takala (Dalian, China). Amplified DNA was purified by PCR purification Kit and plasmid DNA was purified from *E. coli* by SanPrep Column Plasmid Mini-Preps Kit (Sangon Biotech, Shanghai, China). Primers and nucleotide sequences were confirmed by Sangon Biotech (Shanghai, China).

### Recombinant protein expression and purification

*Escherichia coli* BL21-Z cultures harboring recombinant plasmids were grown overnight in tubes at 37 °C and at 200 rpm in LB medium containing ampicillin (100 μg/mL). 0.5 mL of overnight cultures were transferred into 50 mL of fresh LB medium in 250 mL flasks, and grown at 37 °C and at 200 rpm until OD_600_ reached 0.8. Then, isopropyl-β-d-thiogalactopyranoside (IPTG) was added at a final concentration of 1 mM for THK and GCK, 200 μM for IPK and 50 μM for both FAR and ADHE2. The cell cultures were incubated for an additional 20 h at 18 °C and at 160 rpm. The cells were harvested by centrifugation, and resuspended and washed with a buffer (50 mM Tris–HCl, 5 mM imidazole, pH 8.0). The cells were lysed by One Shot cell disruptor (Constant Systems Ltd, United Kingdom) at 3.5 × 10^7^ psi and cell debris was removed by centrifugation at 13,000 rpm for 30 min. The soluble fraction was used for His-tagged purification by Ni-nitrilotriacetic acid (NTA) resin (Pointbio, Shanghai, China). Non-specifically bound proteins were washed out with a buffer (50 mM Tris–HCl, 10 mM MgCl_2_, 20 mM KCl, 30 mM imidazole, pH 8.0), while bound His-tagged proteins were eluted with elution buffer (50 mM Tris–HCl, 10 mM MgCl_2_, 20 mM KCl, 200 mM imidazole, pH 8.0). To remove imidazole and concentrate protein, the eluted solution was centrifuged through a centrifugal filter with a molecular cutoff of 10 kDa (Millipore, Billerica, MA) and purified protein was verified by 12% SDS-PAGE followed by Coomassie Blue staining. The concentration of protein was determined according to Modified BCA Protein Assay Kit (Sangon Biotech, Shanghai, China).

### Characterization of kinases

To characterize the optimal temperature of THK and IPK towards crotonol and crotyl monophosphate, a total reaction volume of 100 μL contained 500 μM crotonol or 500 μM crotyl monophosphate, 5 mM ATP and 0.5 mg/mL THK or 0.02 mg/mL IPK, 10 mM MgCl_2_ and 20 mM KCl in 50 mM Tris–HCl buffer with pH 7.5. For THK, the reaction was performed at temperatures of 29.5 °C, 32 °C, 34.5 °C, 37 °C, 39.5 °C, 42 °C and 44.5 °C for 1 h, and for IPK the reaction was carried out at temperatures of 29.5 °C, 32 °C, 34.5 °C, 37 °C, 39.5 °C, 42 °C and 44.5 °C for 20 min.

For determining the optimal pH of THK on crotonol, a total reaction volume of 100 μL contained 500 μM crotonol, 5 mM ATP, 0.5 mg/mL THK, 10 mM MgCl_2_ and 20 mM KCl in either 50 mM sodium phosphate buffer with pH 6.0 and 6.5 or 50 mM Tris–HCl buffer with pH 7.0, 7.5, 8.0, 8.5 and 9.0. The reaction was performed at 39.5 °C for 1 h. To evaluate the optimal pH of IPK on crotyl monophosphate, the 100 μL reaction contained 500 μM crotyl monophosphate, 5 mM ATP, 0.02 mg/mL IPK, 10 mM MgCl_2_ and 20 mM KCl in either 50 mM sodium phosphate buffer at pH 6.0 and 6.5 or 50 mM Tris–HCl buffer at pH 7.0, 7.5, 8.0, 8.5 or 9.0. The reaction was performed at 39.5 °C for 20 min. All enzymatic reactions were terminated by adding 500 μL cold methanol.

The activity of IPK was determined through coupling the release of ADP with NADH oxidation by pyruvate kinase/lactate dehydrogenase (PK/LDH) [26]. Briefly, enzymatic assays were performed at the optimized condition with the addition of 0.2 mM NADH, 0.5 mM phosphoenolpyruvate, 5 mM ATP and 20 μL aqueous glycerol solution of PK/LDH (Sigma-Aldrich, MO, USA). NADH consumption was measured at 340 nm at 10 s intervals using a UV–visible spectrophotometer (Genesys10S, CA, USA). One unit of IPK activity corresponds to the production of 1 μmol NADH per minute. To measure the activity of THK, the crotyl monophosphate was monitored by LC–MS as described below. One unit of THK activity was defined as the amount of enzyme that catalyzed the formation of 1 μM crotyl monophosphate per minute.

### Kinetics of THK, IPK, FAR and ADHE2

The kinetics of each enzyme was processed via a proportional weighted fit using a nonlinear regression analysis program based on Michaelis–Menten enzyme kinetics. For THK, a total reaction volume of 100 μL contained 5 mM ATP, 0.5 mg/mL THK, 10 mM MgCl_2_ and 20 mM KCl in 50 mM Tris–HCl buffer (pH 8.0) at 39.5 °C. For IPK, the 100 μL reaction contained 5 mM ATP, 0.02 mg/mL IPK, 10 mM MgCl_2_ and 20 mM KCl in 50 mM Tris–HCl buffer (pH 7.5) at 39.5 °C. For FAR,the 100 μL reaction contained 5 mM NADPH, 0.5 mg/mL FAR, 50 mM NaCl_2_ in 50 mM Tris–HCl buffer (pH 7.5) at 37 °C [27]. For ADHE2, the 200 μL reaction contained 0.4 mM NADH, 0.4 mg/mL ADHE2, 1 mM dithiothreitol in 100 mM Tris–HCl buffer (pH 7.5) at 37 °C [28]. The kinetic parameters of THK and IPK were determined when crotonol and crotyl monophosphate were added in a concentration range of 0.1 to 30 mM and 0.062 to 3.0 mM, respectively. The kinetic parameters of FAR and ADHE2 were determined when crotonyl-CoA were added in a concentration range of 0.5 to 13 mM and 0.2 to 8 mM, respectively. To determine the activity of FAR and ADHE2, NADPH and NADH consumptions were measured at 340 nm at 10 s intervals using a UV–visible spectrophotometer [27, 28]. One unit of FAR and ADHE2 activity corresponds to the consumption of 10 nmol NADPH and 10 nmol NADH per minute, respectively.

### Extraction and measurement of crotyl monophosphate and crotyl diphosphate

20 mL of samples at the late of exponential phase (OD_600_ = 1.2 ± 0.1) were rapidly harvested by vacuum filtration using MILLEX-GP PES membrane filters (0.22 μm, 33 mm) and quickly washed with culture medium [29]. Extraction of crotyl monophosphate and crotyl diphosphate was performed as described previously for measuring sugar phosphates in *M. extorquens* AM1 [30]. Briefly, 10 mL of boiling water was added to a given sample and incubated at 100 °C for 10 min. The extracted cell suspension was cooled on ice for 5 min, and then cell debris was removed by centrifugation at 5000 rpm for 5 min. The cell-free extract was centrifuged at 14,000 rpm for 8 min. The supernatant was dried in a rotational vacuum concentrator (Christ, Osterode, Germany) and stored at − 80 °C for further use. For LC–MS analysis, each dried sample was dissolved in 100 μL of purified water. The sample analysis was carried out on an Agilent LC-QQQ-MS system (Agilent 1290 Infnity-6460, Agilent Technologies, Santa Clara, CA, USA). Multiple reaction monitoring (MRM) precursor/product ion pairs were crotyl monophosphate (ESI—m/z 151.0 to m/z 79.0) and crotyl diphosphate (ESI—m/z 231.0 to m/z 79.0). The sample was separated on a Luna^®^ NH_2_ column (150 × 2 mm, 3 μm; Phenomenex, CA, USA). The mobile phase A was acetonitrile/water (5:95 v/v) with 20 mM ammonium acetate and 40 mM ammonium hydroxide. The mobile phase B was acetonitrile/water (95:5 v/v). The linear gradient was as following: 0–2 min, 90% B; 2–22 min, 90–5% B; 22–24 min, 5% B; 24–25 min, 5–90% B; 25–30 min, 90% B. The total run time was 30 min at 0.3 mL/min. The injection volume was 3 μL. All of the peaks were integrated by Qualitative Analysis B.06.00 software. The data were presented as the mean of three independent biological replicates.

### Construction of the *thiM* mutant library and a high-throughput screening

To construct the *thiM* mutants, *thiM* was amplified with primers as shown in Additional file 1: Table S1. The error-prone PCR system contained 0.1 μg of DNA template, 0.2 mM of each primer, 2.5 U of rTaq DNA polymerase, 10× PCR buffer, 0.2 mM dNTP, 0.6 mM dGTP and 3.5 mM Mg^2+^ in a total volume of 50 μL. PCR was performed with a denaturation step at 94 °C for 1 min followed by 30 cycles of 10 s at 98 °C, 1 min at 58.5 °C, and 48 s at 72 °C. The PCR products were cloned into *Bam*HI and *Sac*I sites of pCM80, and then transformed into *M. extorquens* AM1. The recombinant *M. extorquens* AM1 was spread on agar plates and each single clone was picked into tubes containing 3 mL media with either succinate or methanol as carbon source. 200 μM of crotonol was added into the medium when the cell density reached OD_600_ of 0.6. After 8 h of cultivation, the supernatants were harvested by centrifugation and 200 μL was immediately transferred into 96-well plates. Potassium permanganate was added into each well at the final concentration of 200 μM, and the plates were incubated at 30 °C and at 150 rpm for 3 min. After that, 96-well plates were placed in a Spectramax M3 Multi-Mode microplate reader (Molecular Device, CA, USA) to detect the absorbance at 490 nm.

### Saturation mutagenesis

The site-directed *thiM* mutants were generated using a Mutagenesis Kit purchased from Sangon Biotech Ltd. (Shanghai, China). The primers used were listed in the Additional file 1: Table S1. The PCR program was performed according to the Kit protocol. *E. coli* BL21-Z harboring wild-type THK and 19 THK variants were cultured to measure the production of crotyl monophosphate from crotonol. Briefly, 50 mL of cell extracts of *E. coli* BL21-Z were concentrated via a centrifugal filter with a molecular cutoff of 10 kDa (Millipore, Billerica, MA) to 2 mL. This crude enzymatic assay was carried out under the following conditions: 500 μL of reaction mixture contained 50 mM Tris–HCl (pH 8.0), 10 mM MgCl_2_, 20 mM KCl, 5 mM ATP, 5 mM (360 μg/mL) crotonol, 1 mM dithiothreitol, 5% glycerol (v/v) and cell extract. The amount of cell extract was added according to semi-quantification of the band intensity of THK (0.5 mg/mL) by Adobe Photoshop CS 6.0. Crotyl monophosphate was analyzed by LC–MS as described above.

### Homology modeling of hydroxyethylthiazole kinase

To explore the structure of the complex between THK and the substrate crotonol, three-dimensional structure models were constructed using the program Swiss-Model (http://swissmodel.expasy.org/), followed by enzyme-ligand docking using the AutoDockVina program [31]. The structures of THK were modeled using a complex structure template including ATP (pdb id: 1esq). The structure model was subjected to energy minimization using the Swiss-PdbViewer. Afterwards, the docking studies were run with crotonol as ligand and the built THK structure models as receptor. The crotonol structure file (ligand) was retrieved from ZINC site [32]. Docking cluster analysis was performed in the AutoDockVina program environment, and characterized by binding energy. Establishment of eventual complex structural model was based on the energy minimization. The built complex structural analysis was done using Pymol software [33]. The mutation at the specific amino acid site was also introduced using this software, which allowed exploration of the spatial and molecular interactions among amino acids.

### Crude enzymatic assay of *M. extorquens* AM1

For constructing the sequential reactions in *M. extorquens* AM1, DNA fragments of *thiM* and *MTH_47* and the fragments of *adhE2*, *thiM* and *MTH_47* were assembled into the *Bam*HI–*Sac*I restriction sites of pCM80 plasmid under the *mxaF* promoter to obtain pCB1 plasmid and pCB3 plasmid. Cell extracts of recombinants of *M. extorquens* AM1 were generated as described previously with slight modifications [15, 34]. Briefly, 50 mL of cells at the late exponential phase (OD_600_ = 1.2 ± 0.1) were harvested and resuspended in 7 mL of buffer (50 mM Tris–HCl, 10 mM MgCl_2_, 20 mM KCl, pH 8.0). Crude cell extracts were obtained by passing the cells through One Shot cell disruptor at 3.8 × 10^7^ psi. Crude cell extracts were concentrated to 1 mL via centrifugal filter as described above. For detecting the production of crotonol, crotyl monophosphate and crotyl diphosphate from crotonyl-CoA, 500 μL of the reaction mixture contained 50 mM Tris–HCl (pH 8.0), 10 mM MgCl_2_, 20 mM KCl, 4 mM NADH, 5 mM ATP, 1 mM dithiothreitol, 5% glycerol (v/v), and 0.8 mg cell extracts. The enzymatic reaction was started by adding 4 mM crotonyl-CoA into the reaction mixture. The reaction was stopped by adding 2 mM HCl at 4 h. The samples were extracted by 500 μL dichloromethane, and the mixtures were vortexed for 5 min and then centrifuged at 5000 rpm for 10 min to separate the aqueous phase and dichloromethane. Crotonol was analyzed by GCMS-QP2020 system (Shimadzu, Kyoto, Japan) equipped with a Rtx-5MS column (30 m × 0.25 mm × 0.25 μm) via the auto-sampler. 1 μL samples were analyzed with the following program: set initial temperature at 40 °C for 4 min, ramped to 250 °C at 40 °C/min, maintained at 250 °C for 5 min. The ion source temperature was set to 250 °C. GC–MS data were processed using GCMS solution software. For measuring the production of crotyl diphosphate from crotonol, 500 μL of the reaction mixture contained 50 mM Tris–HCl (pH 8.0), 10 mM MgCl_2_, 20 mM KCl, 5 mM ATP, 1 mM dithiothreitol, 5% glycerol (v/v) and 0.3 mg cell extracts. The enzymatic reaction was started by adding 0.2 mM crotonol into the reaction mixture. At time points (2, 4, 6 and 8 h), the reaction was stopped by adding 2.5 mL cold methanol. The samples were centrifuged at 13,000 rpm for 30 min to remove precipitated protein. Crotyl monophosphate and crotyl diphosphate were analyzed by LC–MS as the described above.

## Results and discussion

### Phosphorylation of crotonol into crotyl monophosphate

**Fig. 2:**
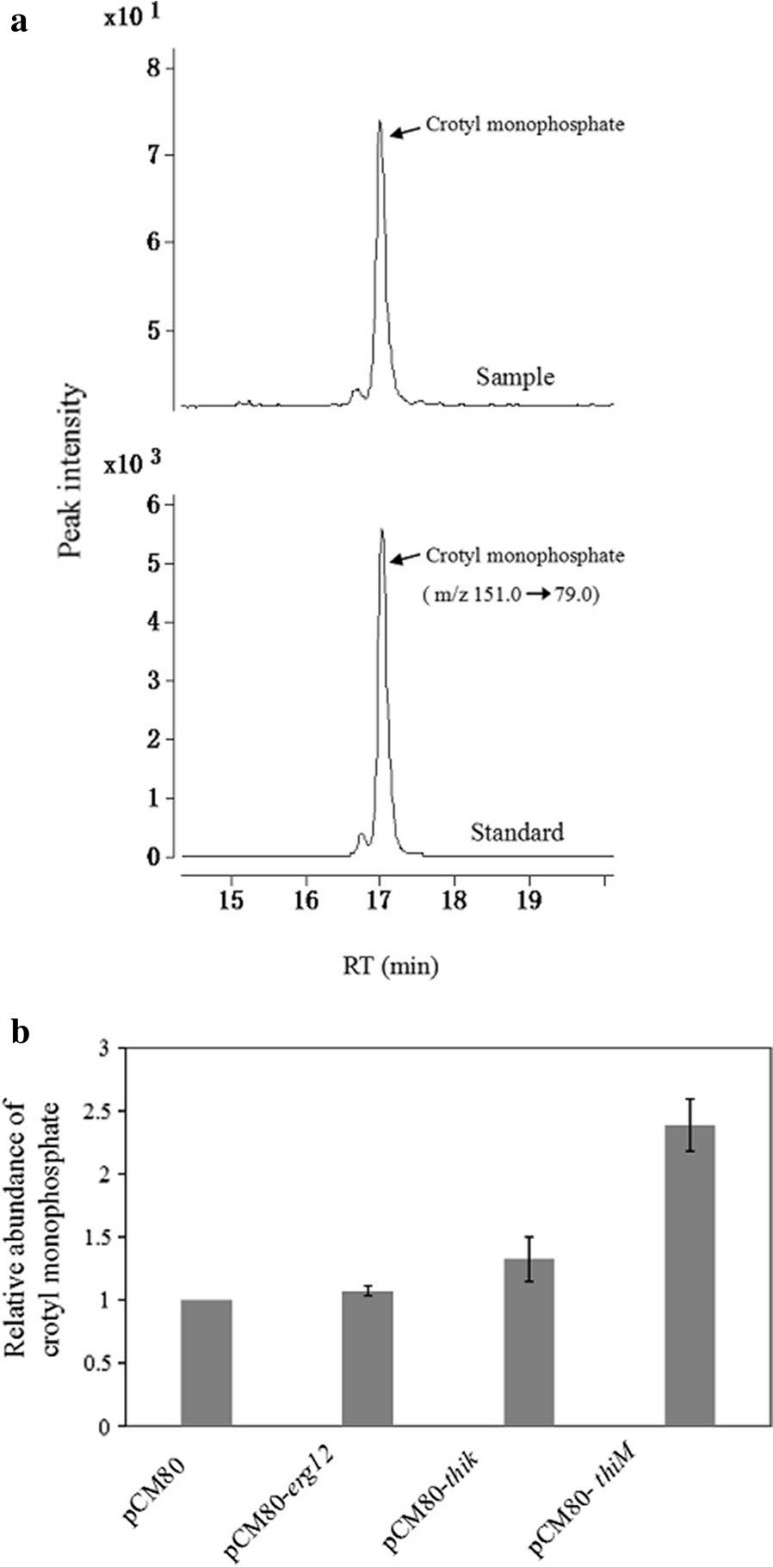
The production of crotyl monophosphate with crotonol feeding by *M. extorquens* AM1 expressing various kinase genes. **a** The extracted ion chromatogram (EIC) of crotyl monophosphate is analyzed by LC–MS in *M. extorquens* AM1 expressing *erg*12. Lower chromatogram is a standard of crotyl monophosphate. **b** crotyl monophosphate production in recombinant *M. extorquens* AM1. The y axis is the ratio of crotyl monophosphate between *M. extorquens* AM1 expressing kinase genes to the control strain carrying empty plasmid pCM80. The average value for the control strain is set to 1. Data represent mean and standard deviations calculated from three biological replicates

The direct phosphorylation of crotonol into crotyl monophosphate has no precedent in the published biochemical literature, but mevalonate kinase (MK) has been well characterized to phosphorylate mevalonate to mevalonate 5-phosphate in the isoprene biosynthesis pathway (Fig. 1) [35]. We first expressed the *erg12* gene encoding MK from *Saccharomyces cerevisiae* in *M. extorquens* AM1 and detected crotyl monophosphate in the culture after the addition of 1 mM crotonol. Intracellular crotyl monophosphate was quantified by LC–MS (Fig. 2a). As shown in Fig. 2b, crotyl monophosphate was detected at a low level slightly above the control. Then, two other types of kinases, i.e. hydroxyethylthiazole kinase (THK) and thiamine kinase (TK), which catalyzed 4-methyl-5-(2-hydroxyethyl)thiazole to 4-methyl-5-(2-phosphonooxyethyl)thiazole and thiamine to thiamine phosphate, respectively, were evaluated for phosphorylation of crotonol. A *thiM* gene encoding THK from *Escherichia coli* and a *thiK* gene encoding TK from *Salmonella enterica* were respectively introduced into *M. extorquens* AM1, and the resultant strains produced 2.4-fold and 1.3-fold higher crotyl monophosphate, respectively, than the control (Fig. 2b). This result indicated that THK was the most suitable kinase among three candidates for phosphorylating crotonol to crotyl monophosphate in *M. extorquens* AM1. Interestingly, crotyl monophosphate was also detected in the control, suggesting wild-type *M. extorquens* AM1 may contain one or more native enzymes capable of phosphorylating crotonol. One of the potential enzymes is the glycerate kinase encoded by the *gck* gene in the serine cycle, which converts d-glycerate to 2-phospho-d-glycerate (2-PGA) [36]. Since this enzyme is the only kinase in central metabolism and has relatively high activity compared to other enzymes involved in the serine cycle and EMC pathway [37], we tested whether purified glycerate kinase could catalyze crotonol phosphorylation. The enzymatic assay showed that glycerate kinase in *M. extorquens* AM1 was able to phosphorylate crotonol with an activity of 0.06 U/mg using 0.5 mM crotonol as substrate (Additional file 2: Fig. S1).

Next, THK was purified and confirmed by SDS-PAGE gel (Additional file 2: Fig. S2), showing the expected molecular weight of 43 kDa including the TRX tag. The conversion efficiency from crotonol into crotyl monophosphate was evaluated in the range between 29.5 and 44.5 °C and the optimal temperature was determined to be 39.5 °C, at which THK generated 1.8-fold and 1.2-fold higher crotyl monophosphate than at 29.5 °C and 44.5 °C respectively (Additional file 2: Fig. S3a). The optimal activities of THK were also determined to be from pH 8.0 to 9.0 in 50 mM Tris–HCl, showing a broad optimum occurred in that range (Additional file 2: Fig. S3b). The kinetic behavior of THK towards crotonol was further characterized at this optimized condition. In vitro enzymatic activity was then assayed by measuring the production of crotyl monophosphate along a time course. The values of *K*_m_ and *k*_*cat*_ were determined to be 8.35 mM and 1.24 s^−1^ (Additional file 2: Fig. S4 and Table 2). The specific activity of THK for 0.5 mM crotonol was 0.1 U/mg, which was 300-fold lower than that for the native substrate 4-methyl-5-(2-hydroxyethyl)thiazole at the same concentration [38]. This result suggested that the activity of THK needed to be further improved through protein engineering in order to supply more crotyl monophosphate for the subsequent reaction.

**Table 2:** Kinetic parameters of THK, THK^M82V^, IPK, FAR and ADHE2

Enzymes	Sources	Substrates	*K*_m_ (mM)	*k*_cat_ (s^−1^)	*k*_cat_/*K*_m_ (mM^−1^ s^−1^)	*V*_max_ (μmol/min/mg)
THK	*E. coli*	Crotonol	8.35 ± 2.24	1.24 ± 0.26	0.15 ± 0.01	0.86 ± 0.18
THK^M82V^	*E. coli*	Crotonol	4.79 ± 0.51	8.58 ± 0.31	1.80 ± 0.13	5.97 ± 0.21
IPK	*M. thermautotrophicus*	Crotyl-monophosphate	1.28 ± 0.50	153.14 ± 18.70	127.94 ± 30.34	77.83 ± 9.5
FAR	*H. chejuensis*	Crotonyl-CoA	3.22 ± 0.07	0.015 ± 0.001	0.005 ± 0.001	0.030 ± 0.002
ADHE2	*C. acetobutylicum*	Crotonyl-CoA	2.34 ± 0.28	1.15 ± 0.27	0.49 ± 0.04	0.25 ± 0.06

### Phosphorylation of crotyl monophosphate into crotyl diphosphate

**Fig. 3:**
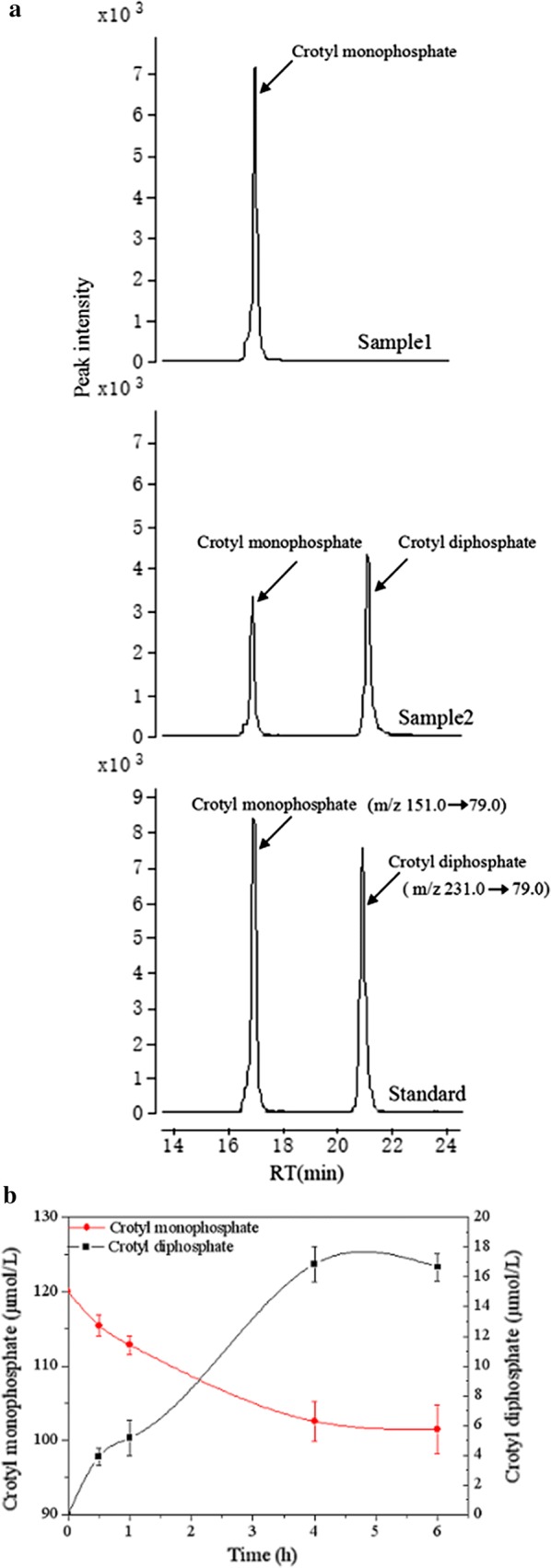
In vitro enzymatic assay detecting crotyl diphosphate in *M. extorquens* AM1 expressing the IPK gene. **a** The EIC of crotyl monophosphate analyzed by LC–MS in the control assay without the addition of crude enzyme IPK (top). The EIC of crotyl monophosphate and crotyl diphosphate in the crude enzyme assay after 4 h (middle). Standards of crotyl monophosphate and crotyl diphosphate (bottom). **b** The increase of in vitro crotyl diphosphate and decrease of crotyl monophosphate in a time course. Data represent mean and standard deviations calculated from three biological replicates

Similar to bioconversion of crotonol into crotyl monophosphate, no enzyme in nature has been reported to be able to catalyze crotyl monophosphate to crotyl diphosphate. Initially, a phosphomevalonate kinase (PMK) described to convert mevalonate-5-phosphate to mevalonate-5-pyrophosphate was selected for evaluation (Fig. 1). We also identified another kinase (isopentenyl phosphate kinase, IPK) from *Methanothermobacter thermautotrophicus* that was able to catalyze a phosphorylation from 3-butenyl phosphate (BEP) to 3-butenyl diphosphate [39]. The structures of BEP and crotyl diphosphate are quite similar and the only difference is the location of the carbon–carbon double bond. The double bond of crotyl monophosphate is between the position of C_2_ and C_3_ whereas the double bond of BEP is between C_3_ and C_4_. Additionally, IPK from *M. thermautotrophicus* or *Thermoplasma acidophilum* was demonstrated to be promiscuous over a broad range of substrates, such as dimethylallyl phosphate, isopentenyl thiolophosphate, 1-butyl phosphate, 3-buten-1-yl phosphate, and geranyl phosphate [39]. Therefore, we expressed the IPK gene in *M. extorquens* AM1 and set up a crude enzymatic assay to evaluate the conversion of crotyl monophosphate. As shown in extracted ion chromatograms, crotyl diphosphate was found to be accumulated after the addition of crotyl monophosphate (Fig. 3a). Accordingly, 3.8 μM of crotyl diphosphate was detected at 0.5 h and increased to 16.6 μM at 4 h after incubation (Fig. 3b). Purified IPK was analyzed by SDS-PAGE, and shown to have a molecular mass of 44 kDa consistent with calculated molecular weight based on amino acid sequence (Additional file 2: Fig. S2). Initial reaction rates of IPK were determined by an assay in which ADP production was coupled to consumption of NADH within 10 min [26]. The optimal temperature and pH of IPK was identified to be 39.5 °C and 7.5 (Additional file 2: Fig. S3). Under the optimal reaction conditions, the kinetic values of *K*_m_ and *k*_cat_ for crotyl monophosphate were measured to be 1.28 mM and 153.14 s^−1^, respectively (Additional file 2: Fig. S4 and Table 2).

### High-throughput screening approach for discovering high activity of THK variants

In order to improve the catalytic efficiency of THK, error-prone PCR libraries were screened with a high-throughput screening (HTS) method to identify a mutated variant with increased enzyme activity. First, a HTS colorimetric assay was developed based on a pink–purple colored agent (potassium permanganate), which loses its color when reduced by crotonol. Thus, the level of residual crotonol after introduction of THK variants can be evaluated by the reduction of potassium permanganate calculated from the absorbance change at 490 nm.

**Fig. 4:**
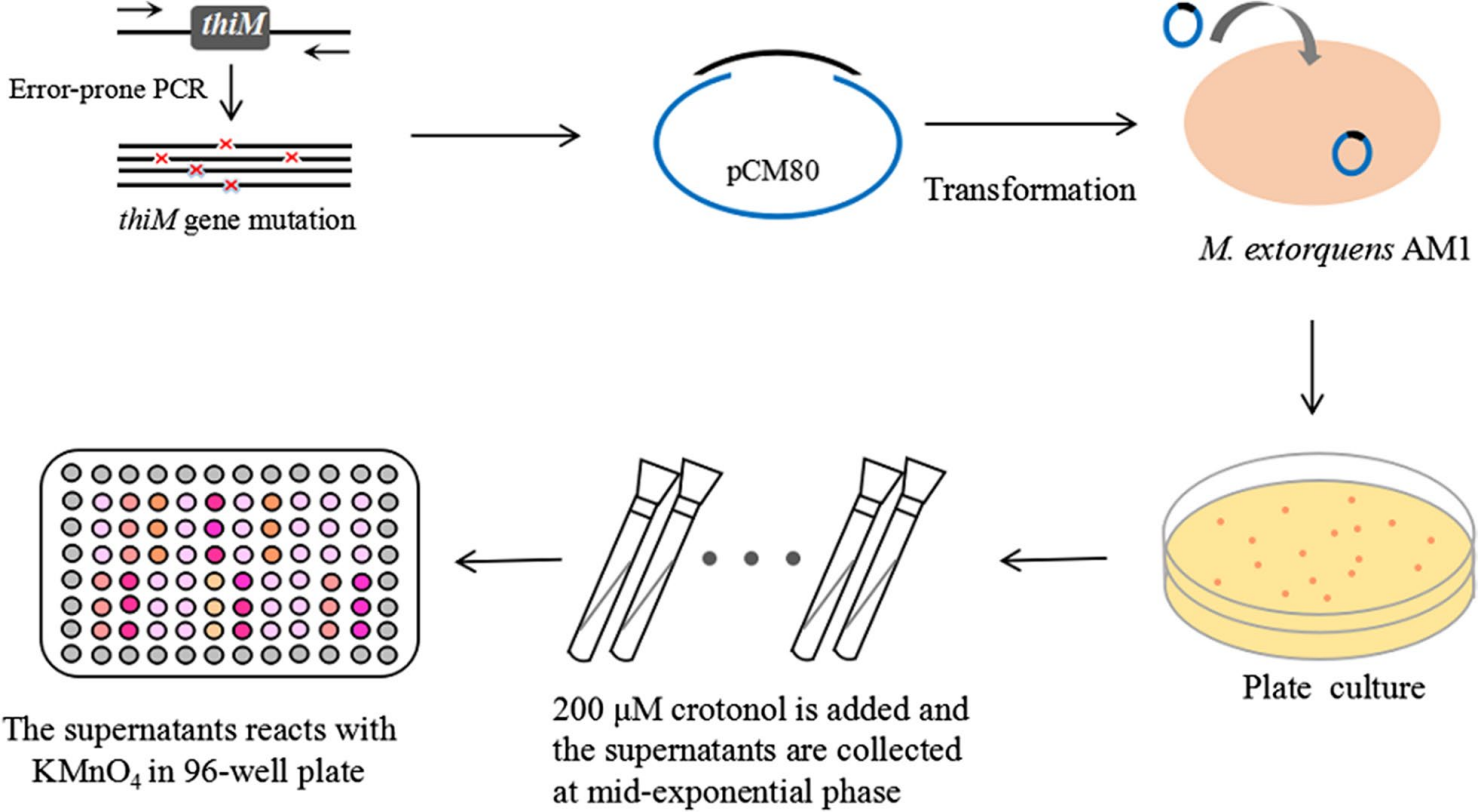
Flowchart of random mutagenesis of *thiM* gene and high-throughput screening assay

The assay development work was carried out with *M. extorquens* AM1 grown on methanol or succinate as the sole carbon source. When *M. extorquens* AM1 was grown on succinate in the presence of crotonol, the colorimetric assay showed a linear correlation (R^2^ of 0.9677) of crotonol decrease and absorbance increase in the range between 0.16 and 0.2 mM of crotonol (Additional file 2: Fig. S5). For operational convenience, we also tried to grow single colonies of mutant strains in 96-well plates. However, the strains stopped growth at an OD_600_ of about 0.15. This phenomenon was in line with the previous report that the growth curves of *M. extorquens* AM1 in 96-well plates had large deviations in the exponential phase [40]. Therefore, the mutant strains were pre-grown in tubes and then the supernatants were transferred to 96-well plates for OD readout in a high-throughput way (Fig. 4). In addition, we measured the level of extracellular crotyl monophosphate in *M. extorquens* AM1 culture to determine whether the produced crotyl monophosphate could be excreted to interfere with the colorimetric assay. No crotyl monophosphate was detected, thereby eliminating the interference of crotyl monophosphate on the screening.

### Improving THK activity by directed evolution

**Fig. 5:**
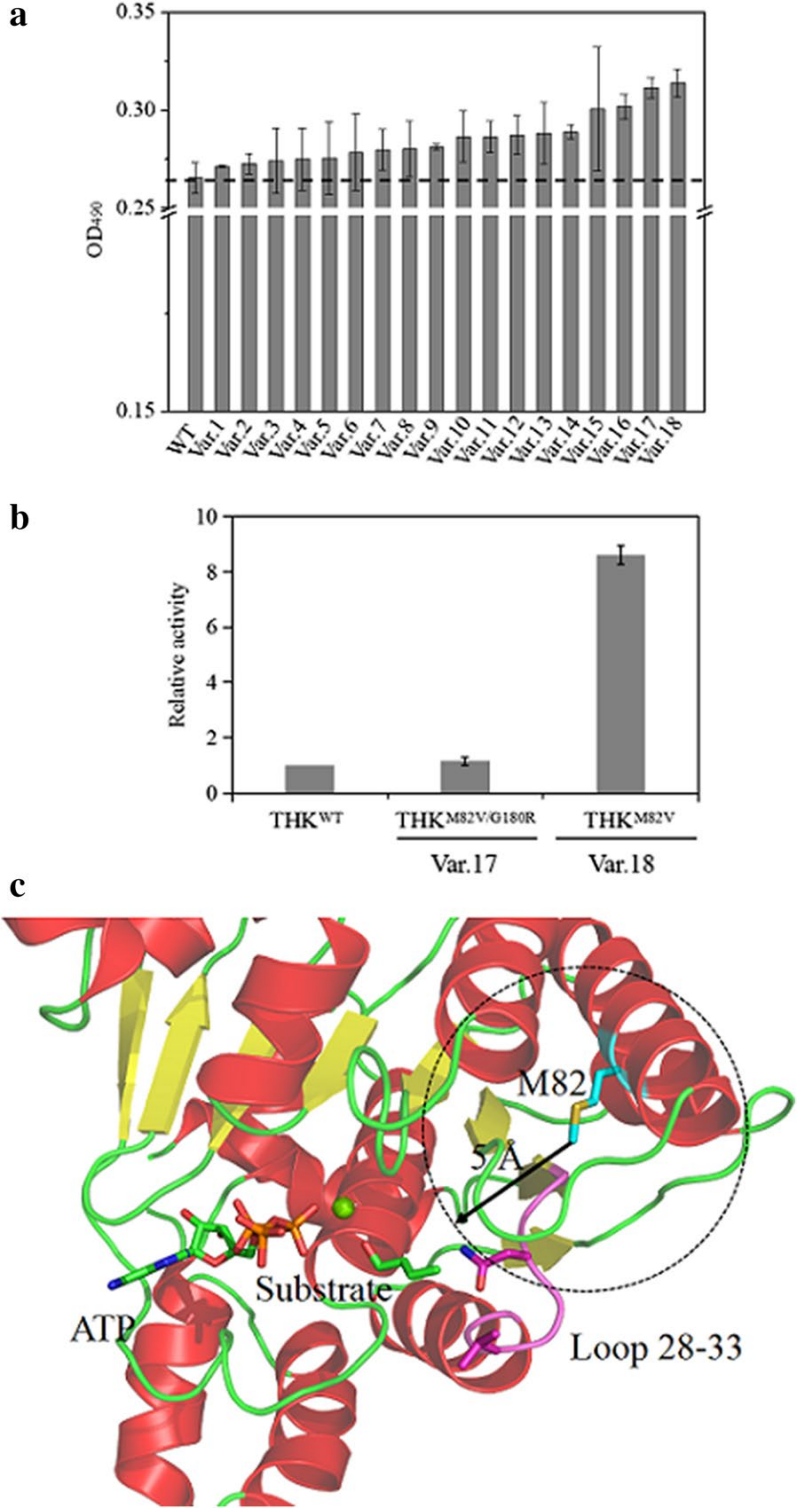
Identification of high active THK variants and molecular docking of wild-type THK and THK^M82V^ with crotonol. **a** Comparison of OD_490_ change between THK variants and wild-type THK. **b** Comparison of specific activities of purified THK^M82V^, THK^M82V/G180R^ and wild-type THK. 4 mM of crotonol is added as substrate. The average value for wild-type THK is set to 1. **c** Molecular docking of wild-type THK and THK^M82V^ with crotonol. Crotonol carbon atoms are colored in green and oxygen atoms in red. ATP carbon atoms are in green, oxygen atoms in red, and phosphate in orange. Loop 28–33 is in purple. The error bars represent the standard deviation of three independent repeats

A random mutagenesis library of *thiM* was made with an average of 1 to 2 point mutations per gene. The generated library was transformed into *M. extorquens* AM1 and a library of around 3100 colonies was created and screened by the HTS for improved enzyme activity. A total of 18 mutants displayed higher enzyme activity than wild-type *thiM* (Fig. 5a), among which two THK variants, i.e. THK^M82V^ and THK^M82V/G180R^, showed the highest activity. In vitro assay indicated that purified THK^M82V^ and THK^M82V/G180R^ had 8.6-fold and 1.2-fold higher activities than wild-type THK (Fig. 5b). Subsequently, a saturation mutagenesis on position 82 was carried out to discover whether there was a more favorable mutation that could further increase the activity of THK. We found that three (M82V, M82I, and M82F) of the 18 variants were highly soluble in the recombinant *E. coli* and M82A, M82P, and M82C were relatively insoluble (Additional file 2: Fig. S6). Enzyme assays with crude extracts from *E. coli* were carried out to detect the production of crotyl monophosphate. None of these variants showed higher crotyl monophosphate compared to THK^M82V^ (Additional file 2: Fig. S6). It has been reported that the activity of a heterologous protein was affected by its solubility [41]. The significant decrease in production of crotyl monophosphate for the variants THK ^M82D^, THK^M82N^, THK^M82E^, THK^M82G^, THK^M82W^ and THK^M82K^ was likely due to insolubility resulting in inactivity as well.

In order to surmise the molecular mechanisms conferring higher enzymatic activity, we conducted a homology modeling analysis. As shown in the modeled structure of THK (Fig. 5c), M82 is adjacent to the significant loop 28–33 (distance < 5 Å) which is part of the substrate-binding pocket. Probably, this impedes binding of smaller substrate molecules and, thus, contributes to the specificity of the enzyme. When M82 was mutated to V82, the above-mentioned hindrance around M82 was relieved, likely improving the catalytic activity to the smaller molecule substrate such as crotonol. As shown in Additional file 2: Fig. S4 and Table 2, the *K*_m_ value of THK^M82V^ was determined to be 4.79 mM, 42% lower than wild-type THK, indicating a higher affinity to crotonol. And the *k*_cat_ value was 8.58 s^−1^, representing a 6.9-fold improvement in turnover rate. As a consequence, the *k*_cat_/*K*_m_ value of THK^M82V^ was 12-fold higher than that of wild-type THK.

### Optimization of THK ^M82V^ and IPK concentrations for in vitro reaction

The optimal amount of IPK and THK^M82V^ required for efficient conversion of crotonol further into crotyl diphosphate in a one-pot reaction was also evaluated. As shown in Additional file 2: Fig. S7a, the production of crotyl monophosphate was increased by 1.4-fold at 8 h when the addition of THK^M82V^ was increased from 0.5 to 1.5 mg/mL, then reached a plateau at 2.0 mg/mL. Thus, 1.5 mg/mL of THK^M82V^ was used for the one-pot reaction system. In Additional file 2: Fig. S7b, the titers of crotyl diphosphate reached 69.7, 87.5, 99.1 and 102.7 μg/mL at 4 h corresponding to 0.25, 0.5, 1.0 and 1.5 mg/mL of IPK, respectively. As the catalytic efficiency of IPK (*k*_cat_/*K*_m_ = 127.94 mM^−1^ s^−1^) was 71-fold higher than that of THK ^M82V^ (*k*_cat_/*K*_m_ = 1.80 mM^−1^ s^−1^), the lower concentration of IPK (0.5 mg/mL) was chosen for one-pot reactions. Moreover, the in vitro reaction was conducted under the optimal temperature and pH for THK^M82V^ in order to enhance the supply of crotyl monophosphate. Notably, the production of crotyl monophosphate and crotyl diphosphate showed a decreasing rate after 2 h for all the tested conditions (Additional file 2: Fig. S7), implying that those kinases were possibly deactivated gradually along with the reaction. We did not find any literature studying the stability of these two kinases [42], but did notice the conversion was significantly decreased in our preliminary experiments when the enzymes were stored in the reaction buffer overnight before usage.

### In vitro reaction to convert crotonol into crotyl diphosphate

**Fig. 6:**
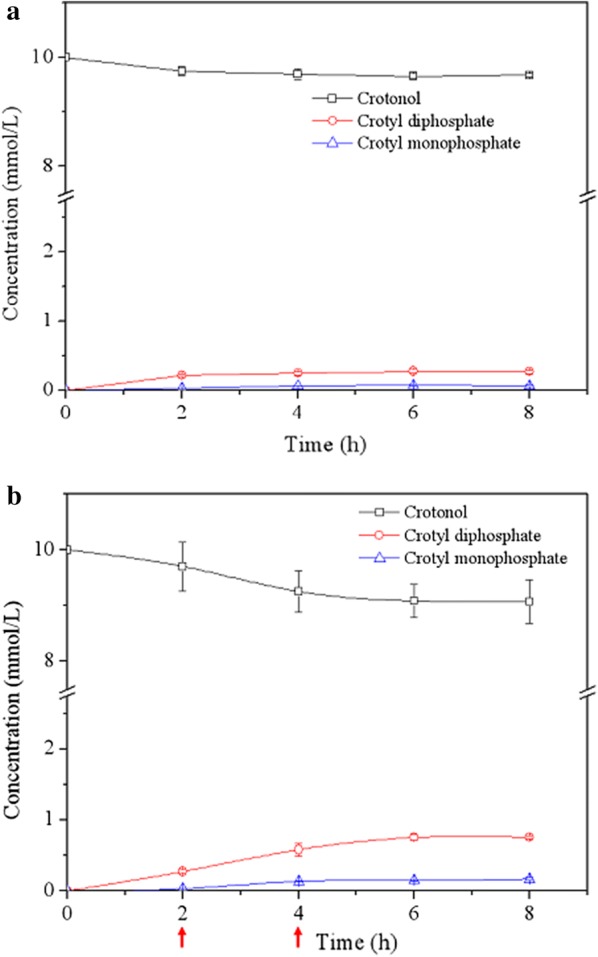
One-pot reaction for producing crotyl diphosphate from crotonol. **a** Crotonol consumption, crotyl monophosphate and crotyl diphosphate production in one-pot reaction system (1.5 mg/mL of THK^M82V^ and 0.5 mg/mL of IPK at 39.5 °C and pH 8.0). **b** One-pot reaction with reloading the kinases of THK^M82V^ and IPK at 2 h and 4 h (red arrow). Data show the mean with error bars indicating standard deviation calculated from three independent biological replicates

The two kinases and crotonol were co-incubated under the aforementioned conditions (i.e. 10 mM of crotonol, 1.5 mg/mL THK^M82V^, 0.5 mg/mL of IPK, 200 μL reaction system, at 39.5 °C and pH 8.0) for production of crotyl diphosphate. As expected, the crotyl diphosphate was produced in a linear manner to 0.22 mM within 2 h, equal to a 2.2% conversion rate from crotonol (Fig. 6a). Beer et al. demonstrated that NADH oxidase was not stable at O_2_ saturated conditions in the one-pot reaction for converting glucuronate to α-ketoglutarate, and this oxidase was added three times resulting in the increase of conversion rate by 4.76-fold [43]. In our case, a similar phenomenon was observed by replenishing THK ^M82V^ and IPK at 2 h and 4 h, which increased the conversion rate by 3.45-fold. Eventually, the titer of crotyl diphosphate was increased to 0.76 mM at 6 h, corresponding to a 7.6% conversion (Fig. 6b). Notably, crotyl monophosphate was barely detected in the whole time course (Fig. 6), suggesting THK was still a rate-limiting step. Future work is possible to significantly increase the activity of THK by a combination of homology modeling established in this work and iterative evolution based on the THK^M82V^.

### Conversion of crotonyl-CoA to crotonol by an aldehyde/alcohol dehydrogenase

**Fig. 7:**
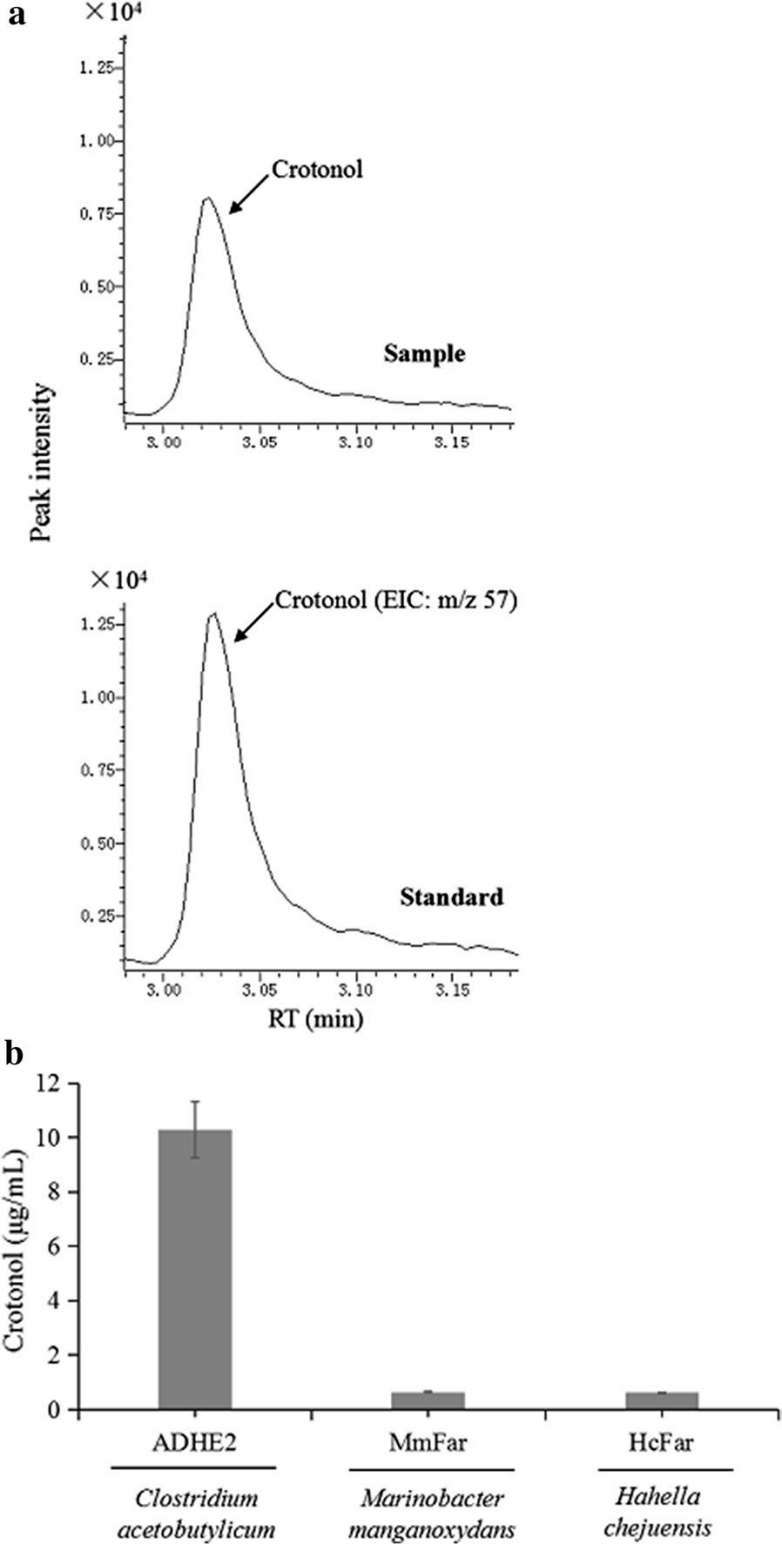
In vitro enzymatic assay detected the production of crotonol catalyzed by purified enzymes. **a** The EIC (m/z 57) of crotonol analyzed by GC–MS. The enzymatic sample was carried out by ADHE2. **b** Comparison of crotonol production among three purified enzymes. The crotonyl-CoA was added as 4 mM (3.34 mg/mL). The assay time was 4 h. Data show the mean with error bars indicating standard deviation calculated from three independent biological replicates

To identify a candidate enzyme for producing crotonol from crotonyl-CoA involved in the EMC pathway, we tested aldehyde/alcohol dehydrogenase (ADHE2) from *C. acetobutylicum* and fatty acyl-CoA reductase (FAR) from *H. chejuensis* and *M. manganoxydans.* ADHE2 has been used to convert short chain acyl-CoA to alcohols in either engineered *M. extorquens* AM1 or *Clostridium* species [16, 44]. FAR is another promiscuous enzyme which has good rates of reduction for longer (C20) and shorter (C8) fatty acyl-CoA groups or longer (C8) and shorter (C2) aldehyde groups to produce various alcohols [27]. We found that purified ADHE2 from *C. acetobutylicum* could reduce crotonyl-CoA to crotonol more efficiently compared with FAR from *H. chejuensis* and *M. manganoxydans* by measuring the production of crotonol on GC–MS (Fig. 7). The purified ADHE2 was confirmed by SDS-PAGE gel (Additional file 2: Fig. S2), showing the expected molecular weight of 109 kDa. The kinetic values of ADHE2 were then determined as *K*_m_ and *k*_cat_ values of 2.34 mM and 1.15 s^−1^ (Additional file 2: Fig. S4, Table 2). This catalytic efficiency towards crotonyl-CoA (*k*_*cat*_/*K*_m_ = 0.49 mM^−1^ s^−1^) was lower than that of ADHE2 towards butanyl-CoA (*k*_*cat*_/*K*_*m*_ = 152 mM^−1^ s^−1^) [45].

### Constructing a heterologous pathway to produce butadiene precursor in *M. extorquens* AM1

**Fig. 8:**
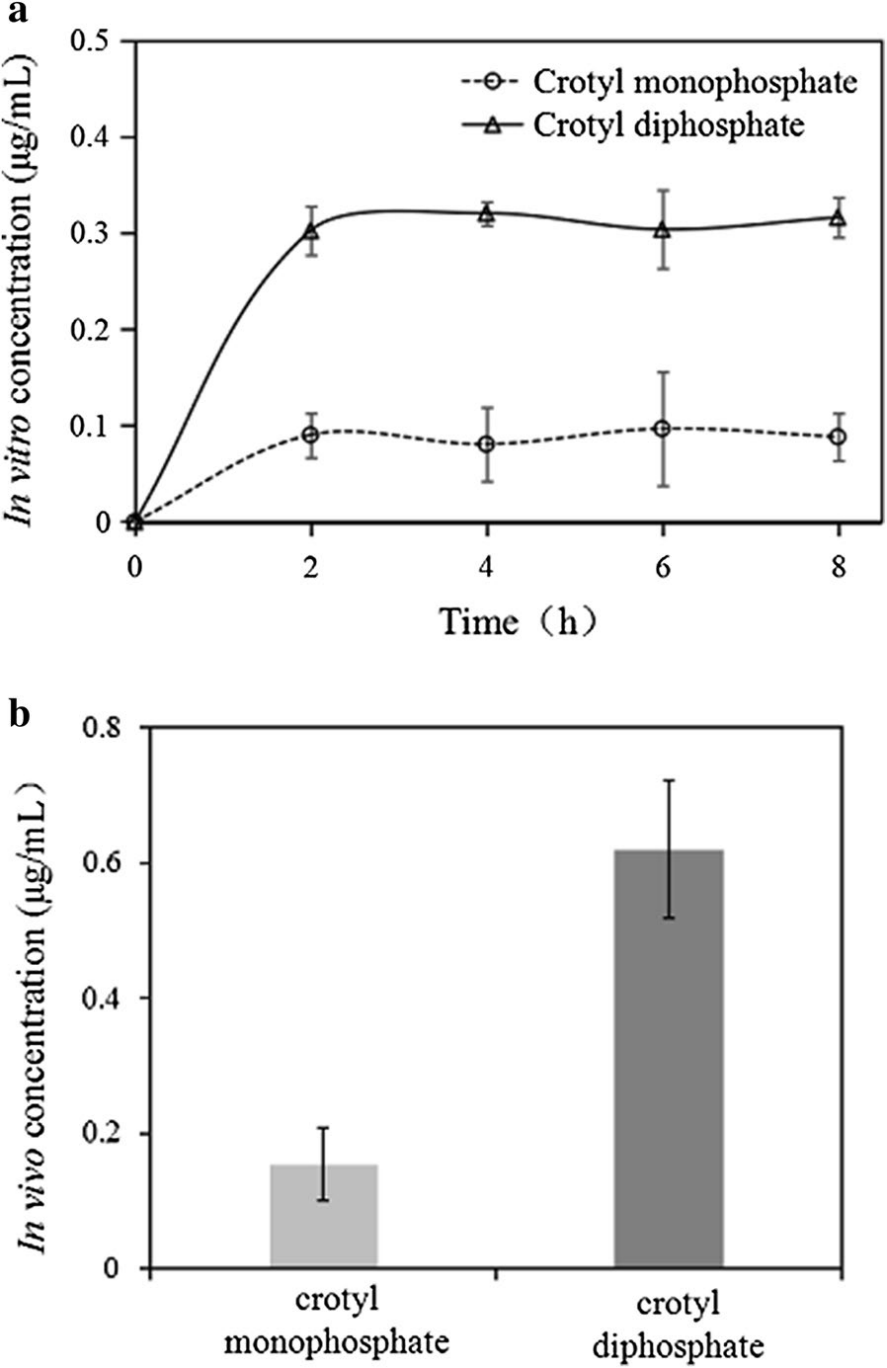
Crotyl diphosphate production in the YCB1 strain expressing *thiM* and IPK genes. **a** In vitro crotyl monophosphate and crotyl diphosphate production. Crotonol was 0.2 mM (14.4 μg/mL) and cell lysates were from the YCB1 strain. **b** In vivo crotyl diphosphate formation in the YCB1 strain. 2 mM crotonol was added into the culture medium when the cell density reached OD_600_ of 0.6. Data show the mean with error bars indicating standard deviation calculated from three independent biological replicates

Following the in vitro reaction, we further expressed *thiM* and the MTH_47 gene (encoding IPK) in *M. extorquens* AM1 and the engineered strain was named YCB1. Cell lysates of YCB1 were incubated with appropriate amounts of ATP and crotonol. As shown in Fig. 8a, 0.34 μg/mL of crotyl diphosphate was detected at 2 h in the engineered strains while the controls lacking THK and IPK did not yield detectable crotyl diphosphate in the presence of crotonol. We also detected the production of crotyl diphosphate in vivo by adding 2 mM of crotonol during inoculation. About 0.60 μg/mL (2.59 μM) of intracellular crotyl diphosphate was accumulated at mid-exponential phase (Fig. 8b), which was comparable to the individual intermediates in assimilation pathways, which ranged from 0.13 to 55.6 μM in *M. extorquens* AM1 grown on methanol [46, 47].

In order to explore the possibility of producing crotyl diphosphate from crotonyl-CoA, the engineered *M. extorquens* AM1 (YCB3 strain) containing the genes for ADHE2, THK^M82V^ and IPK was further constructed and tested for crotyl diphosphate production. However, no crotyl diphosphate was produced by YCB3 during growth on methanol. The engineered *M. extorquens* AM1 (YCB4 strain) containing *adhE2* was also analyzed for crotonol production when OD reached 0.6, 1.2 and 1.4. No crotonol was detected either. One bottleneck was probably insufficient supply of crotonyl-CoA [14, 17]. Thus, we performed a crude enzymatic assay where a high concentration of crotonyl-CoA was added to drive the reaction from crotonyl-CoA to crotyl diphosphate. Trace concentration of crotyl diphosphate was detected, and 0.51 μg/mL of crotonol (7.0 μM) was produced at 4 h. This result indicated that low activity of ADHE2 was one major bottleneck resulting in the lack of reduction of crotonyl-CoA into crotyl alcohol. Just recently, Becher et al. characterized a novel CoA-acylating aldehyde dehydrogenase responsible for prenal (3-methyl-2-butenal) to 3-methylcrotonyl-CoA oxidation [48], which could possibly improve the activity for the first step of reduction of crotyl-CoA to crotonaldehyde. Future direction will be focused on improving catalytic efficiency of crotyl-CoA into crotyl alcohol to realize the production of butadiene precursor from methanol.

## Conclusions

In this work we engineered a metabolic pathway in *M. extorquens* AM1 for converting crotonol into crotyl diphosphate, a direct precursor of butadiene. The pathway contains a hydroxyethylthiazole kinase (THK) from *E. coli* and isopentenyl phosphate kinase (IPK) from *M. thermautotrophicus*. Directed evolution of the rate-limiting THK resulted in a variant (M82V) with the *k*_cat_/*K*_m_ value 12-fold higher than that of wild-type THK. As a consequence, 7.6% of crotonol was converted into crotyl diphosphate at an optimized in vitro condition. Moreover, the pathway of crotonyl-CoA into crotyl diphosphate was constructed in *M. extorquens* AM1. 0.60 μg/mL of intracellular crotyl diphosphate was accumulated at the middle of exponential phase with crotonol feeding, and 0.51 μg/mL of crotonol was produced from crotonyl-CoA in vitro crude enzymatic assay. The engineered *M. extorquens* AM1, however, cannot produce crotyl diphosphate from methanol yet. This was likely because of low activity of ADHE2 towards crotonyl-CoA reduction. In the future, it should be possible to address this issue including protein design and manipulating the flux through the EMC pathway [14, 48, 49]. Although further enzymes and strain optimization are required to make this system industrially relevant, this novel work is the first example for biosynthesis of butadiene precursors. Future work will focus on increasing the activity of ADHE2 in *M. extorquens* AM1 to realize the bioconversion of methanol into economically important product of butadiene.

## Additional files

**Additional file 1: Table S1.** All the primers are used in this work.

**Additional file 2: Fig. S1.** Specific activity of purified glycerate kinase (GCK) towards crotonol. The crotonol is added at 500 μM and GCK is 0.5 mg/mL. The control has no added crotonol. **Fig. S2.** SDS-PAGE analysis of purified THK, THK^M82V^, IPK and FAR. a M1, M2: Protein markers; 1: Purified THK. b M1, M2: Protein markers; 1: Purified THK^M82V^; 2: Purified IPK. c M1: Protein marker, 1: Purified ADHE2. d M1: Protein marker, 1: Purified FAR, originated from *Hahella chejuensis*; **Fig. S3.** Determining the optimal temperature and pH for THK and IPK. a The optimal temperature of THK and IPK. b The optimal pH of THK and IPK. Data represent mean and standard deviations calculated from three biological replicates. **Fig. S4.** Enzymatic kinetics of THK, THK^M82V^, IPK, FAR and ADHE2. a Wild-type THK towards crotonol. b IPK towards crotyl monophosphate. c THK^M82V^ towards crotonol. d FAR towards crotonyl-CoA. e ADHE2 towards crotonyl-CoA Data represent mean and standard deviations calculated from three biological replicates. **Fig. S5.** Development of a high throughput screening method. Wild-type *M. extorquens* AM1 was grown on succinate to mid-exponential phase (OD_600_ = 0.60), then crotonol from 160 to 200 mM was added into the culture medium. The supernatants were then transferred into 96-well plate and potassium permanganate was added at a final concentration of 200 μM. a Color reaction between crotonol and potassium permanganate for 3 min. b The linear correlation between crotonol concentration and OD_490_ value. Data show the mean with error bars indicating standard deviation calculated from three independent biological replicates. **Fig. S6.** The effect of targeted mutation on THK activity. a Activity for mutants of the 82th amino acid of THK. A crude enzymatic assay detects the production of crotyl monophosphate by LC–MS at 1 h. b Crude proteins extracted from *E. coli* are analyzed by SDS-PAGE gel. Data show the mean with error bars indicating standard deviation calculated from three independent biological replicates. **Fig. S7.** The effect of kinase concentrations on the production of crotyl monophosphate and crotyl diphosphate in vitro. **a** Time and concentration curves of crotyl monophosphate with 10 mM (720 μg/mL) crotonol as substrate and catalyzed by loading different concentrations of THK^M82V^. **b** Time and concentration curves of crotyl diphosphate with 4 mM (608 μg/mL) crotyl monophosphate as substrate and catalyzed by adding different concentrations of IPK. Data represent mean and standard deviations calculated from three biological replicates.

## Abbreviations

THK: hydroxyethylthiazole kinase
IPK: isopentenyl phosphate kinase
EMC pathway: ethylmalonyl-CoA pathway
PHB: poly-3-hydroxybutyrate
LB: Luria–Bertani
Tet: tetracycline
FAR: fatty acyl-CoA reductase
ADHE2: aldehyde/alcohol dehydrogenase
GCK: glycerate kinase
IPTG: isopropyl-β-d-thiogalactopyranoside
NTA: Ni-nitrilotriacetic acid
PK: pyruvate kinase
LDH: lactate dehydrogenase
MRM: multiple reaction monitoring
MK: mevalonate kinase
EIC: the extracted ion chromatogram
TK: thiamine kinase
2-PGA: 2-phospho-d-glycerate
PMK: phosphomevalonate kinase
BEP: 3-butenyl phosphate
HTS: high-throughput screening
