# Supplementary material for: Metabolic engineering of Methylobacterium extorquens AM1 for the production of butadiene precursor
Source: Microb Cell Fact. 2018 Dec 20;17:194. doi: 10.1186/s12934-018-1042-4 (PMC6300920; doi:10.1186/s12934-018-1042-4)
Supplement: Supplementary file 1 — Additional file 1: Table S1. All the primers are used in this work. [file 12934_2018_1042_MOESM1_ESM.docx]

**Table S1. All the primers are used in this work.**

| Primers | Sequence (5’–3’) |
| --- | --- |
| *thim*_ *Bam*H I_F | GAGGGATCCATGCAAGTCGACCTGCTGGGTTC |
| *thim*_ *Sac* I _R | GAGGAGCTCTCATGCCTGCACCTCCTGCGTC |
| *thik*_ *Bam*H I _F | GAGGGATCCGTCCGCAGCAACAACAACAAC |
| *thik* _ *Sac* I _R | GAGGAGCTCTCAGCCCTTCATGCGGAG |
| *erg12*_*Bam*H I_F | GAGGGATCCATGTCCCTCCCCTTCCTCACGTC |
| *erg12*_ *Sac* I _R | GAGGAGCTCTCAGGAGGTCCAGGGGAGGTTC |
| MTH_47_ *Bam*H I _ F | GAGGGATCCATGATCATCCTCAAGCTGGGCGGCT |
| MTH_47 _ *Sac* I _ R | GAGGAGCTCTCAGTGCTTGCCGGTGATGCGGGT |
| *gck*_*Bam*H I_F | GAGGGATCCATGACCGCCCCTGCCCCGAC |
| *gck*_*Sac* I_R  *far*_*Bam*H I _F  *far*_ *Hin*dIII _R  *adhe*2_*Bam*H I _F  *adhe*2_ *Hin*dIII_R  *thim*-MTH_47_OL_R  *thim*-MTH_47_OL_F  *adhe*2*-thim*-MTH_47_OL_R  *adhe*2*-thim*-MTH_47_OL_F  M82A-F | GAGGAGCTCTCAACCGACGAGGATGACGCGGCAATC  CTCGGATCCATGAACTACTTCGTCACCGGCGGCAC  GAGAAGCTTTCACCAGTGGATGCCGCGCATG  CTCGGATCCATGAAGGTCACCAACCAGAAG  GAGAAGCTTTCAGAAGGACTTGATGTAGATGTC  GCTTGAGGATGATCATATGGAATTCTCCAATCATGCCTGCACCTCCTGCGTC  GGAGGTGCAGGCATGATTGGAGAATTCCATATGATCATCCTCAAGCTGGGCGGCT  GAACCCAGCAGGTCGACTTGCATATGGAATTCTCCAATTACCAGTGAATTCCCCGCATGATC  GATCATGCGGGGAATTCACTGGTAATTGGAGAATTCCATATGAAGGTCACCAACCAGAAG  CAGCCACGCGCTCAGGCGGCACGTGCTGCCGTTGAGCAAG |
| M82A-R | CTTGCTCAACGGCAGCACGTGCCGCCTGAGCGCGTGGCTG |
| M82C-F | CAGCCACGCGCTCAGGCGTGTCGTGCTGCCGTTGAGCAAG |
| M82C-R | CTTGCTCAACGGCAGCACGACACGCCTGAGCGCGTGGCTG |
| M82D-F  M82D-R  M82E-F  M82E-R  M82F-F  M82F-R  M82G-F  M82G-R  M82H-F  M82H-R  M82I-F  M82I-R  M82K-F  M82K-R  M82L-F  M82L-R  M82N-F  M82N-R  M82P-F  M82P-R  M82Q-F  M82Q-R  M82R-F  M82R-R  M82S-F  M82S-R  M82T-F  M82T-R  M82W-F  M82W-R  M82Y-F  M82Y-R | CAGCCACGCGCTCAGGCGGATCGTGCTGCCGTTGAGCAAG  CTTGCTCAACGGCAGCACGATCCGCCTGAGCGCGTGGCTG  CAGCCACGCGCTCAGGCGGAACGTGCTGCCGTTGAGCAAG  CTTGCTCAACGGCAGCACGTTCCGCCTGAGCGCGTGGCTG  CAGCCACGCGCTCAGGCGTTTCGTGCTGCCGTTGAGCAAG  CTTGCTCAACGGCAGCACGAAACGCCTGAGCGCGTGGCTG  CAGCCACGCGCTCAGGCGGGTCGTGCTGCCGTTGAGCAAG  CTTGCTCAACGGCAGCACGACCCGCCTGAGCGCGTGGCTG  CAGCCACGCGCTCAGGCGCATCGTGCTGCCGTTGAGCAAG  CTTGCTCAACGGCAGCACGATGCGCCTGAGCGCGTGGCTG  CAGCCACGCGCTCAGGCGATTCGTGCTGCCGTTGAGCAAG  CTTGCTCAACGGCAGCACGAATCGCCTGAGCGCGTGGCTG  CAGCCACGCGCTCAGGCGAAACGTGCTGCCGTTGAGCAAG  CTTGCTCAACGGCAGCACGTTTCGCCTGAGCGCGTGGCTG  CAGCCACGCGCTCAGGCGCTGCGTGCTGCCGTTGAGCAAG  CTTGCTCAACGGCAGCACGCAGCGCCTGAGCGCGTGGCTG  CAGCCACGCGCTCAGGCGAATCGTGCTGCCGTTGAGCAAG  CTTGCTCAACGGCAGCACGATTCGCCTGAGCGCGTGGCTG  CAGCCACGCGCTCAGGCGCCCCGTGCTGCCGTTGAGCAAG  CTTGCTCAACGGCAGCACGGGGCGCCTGAGCGCGTGGCTG  CAGCCACGCGCTCAGGCGCAGCGTGCTGCCGTTGAGCAAG  CTTGCTCAACGGCAGCACGCTGCGCCTGAGCGCGTGGCTG  CAGCCACGCGCTCAGGCGCGGCGTGCTGCCGTTGAGCAAG  CTTGCTCAACGGCAGCACGCCGCGCCTGAGCGCGTGGCTG  CAGCCACGCGCTCAGGCGAGCCGTGCTGCCGTTGAGCAAG  CTTGCTCAACGGCAGCACGGCTCGCCTGAGCGCGTGGCTG  CAGCCACGCGCTCAGGCGACCCGTGCTGCCGTTGAGCAAG  CTTGCTCAACGGCAGCACGGGTCGCCTGAGCGCGTGGCTG  CAGCCACGCGCTCAGGCGTGGCGTGCTGCCGTTGAGCAAG  CTTGCTCAACGGCAGCACGCCACGCCTGAGCGCGTGGCTG  CAGCCACGCGCTCAGGCGTATCGTGCTGCCGTTGAGCAAG  CTTGCTCAACGGCAGCACGATACGCCTGAGCGCGTGGCTG |

^*^Underlining indicates the mutation sites.
