# Supplementary material for: Metabolic engineering of Methylobacterium extorquens AM1 for the production of butadiene precursor
Source: Microb Cell Fact. 2018 Dec 20;17:194. doi: 10.1186/s12934-018-1042-4 (PMC6300920; doi:10.1186/s12934-018-1042-4)
Supplement: Supplementary file 2 — Additional file 2: Fig. S1. Specific activity of purified glycerate kinase (GCK) towards crotonol. The crotonol is added at 500 μM and GCK is 0.5 mg/mL. The control has no added crotonol. Fig. S2. SDS-PAGE analysis of purified THK, THKM82V, IPK and FAR. a M1, M2: Protein markers; 1: Purified THK. b M1, M2: Protein markers; 1: Purified THKM82V; 2: Purified IPK. c M1: Protein marker, 1: Purified ADHE2. d M1: Protein marker, 1: Purified FAR, originated from Hahella chejuensis; Fig. S3. Determining the optimal temperature and pH for THK and IPK. a The optimal temperature of THK and IPK. b The optimal pH of THK and IPK. Data represent mean and standard deviations calculated from three biological replicates. Fig. S4. Enzymatic kinetics of THK, THKM82V, IPK, FAR and ADHE2. a Wild-type THK towards crotonol. b IPK towards crotyl monophosphate. c THKM82V towards crotonol. d FAR towards crotonyl-CoA. e ADHE2 towards crotonyl-CoA Data represent mean and standard deviations calculated from three biological replicates. Fig. S5. Development of a high throughput screening method. Wild-type M. extorquens AM1 was grown on succinate to mid-exponential phase (OD600 = 0.60), then crotonol from 160 to 200 mM was added into the culture medium. The supernatants were then transferred into 96-well plate and potassium permanganate was added at a final concentration of 200 μM. a Color reaction between crotonol and potassium permanganate for 3 min. b The linear correlation between crotonol concentration and OD490 value. Data show the mean with error bars indicating standard deviation calculated from three independent biological replicates. Fig. S6. The effect of targeted mutation on THK activity. a Activity for mutants of the 82th amino acid of THK. A crude enzymatic assay detects the production of crotyl monophosphate by LC–MS at 1 h. b Crude proteins extracted from E. coli are analyzed by SDS-PAGE gel. Data show the mean with error bars indicating standard deviation calculated from three inde [file 12934_2018_1042_MOESM2_ESM.docx]

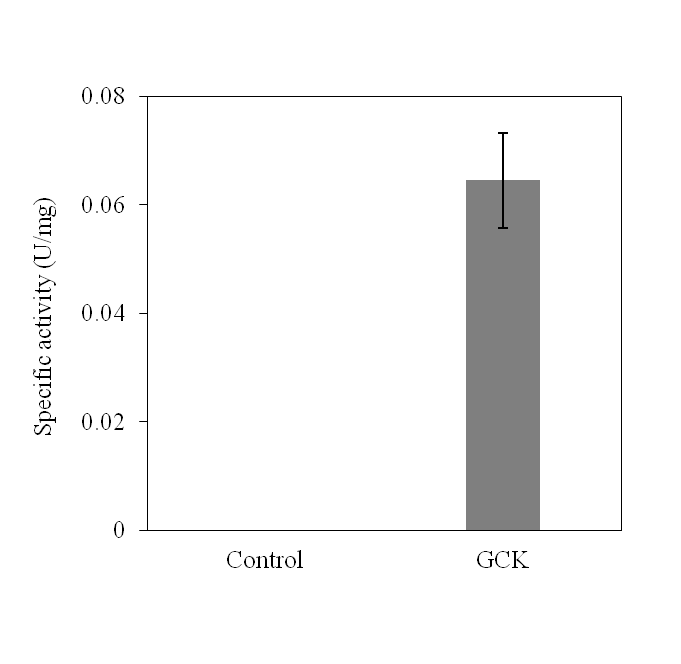


**Fig. S1. Specific activity of purified glycerate kinase (GCK) towards crotonol.**

The crotonol is added at 500 μM and GCK is 0.5 mg/mL. The control has no added crotonol.

**
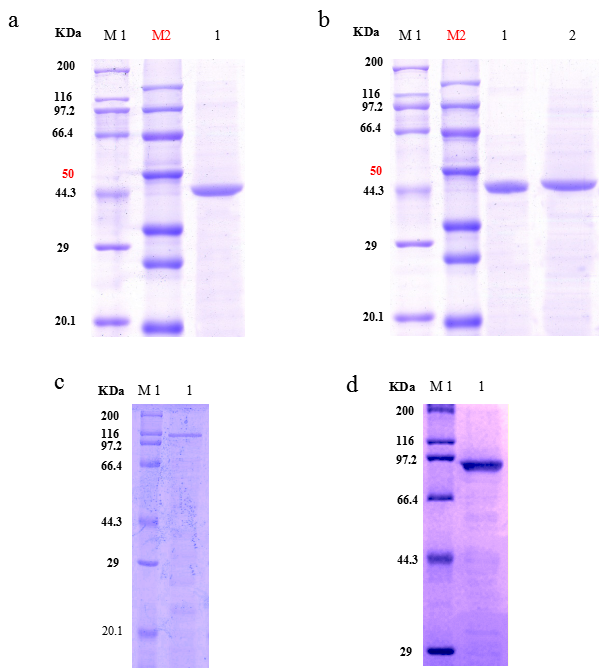
**

**Fig. S2. SDS-PAGE analysis of purified THK, THK^M82V^, IPK and FAR.**

**a** M1, M2: Protein markers; 1: Purified THK.

**b** M1, M2: Protein markers; 1: Purified THK^M82V^; 2: Purified IPK.

**c** M1: Protein marker, 1: Purified ADHE2.

**d** M1: Protein marker, 1: Purified FAR, originated from *Hahella chejuensis*;

**
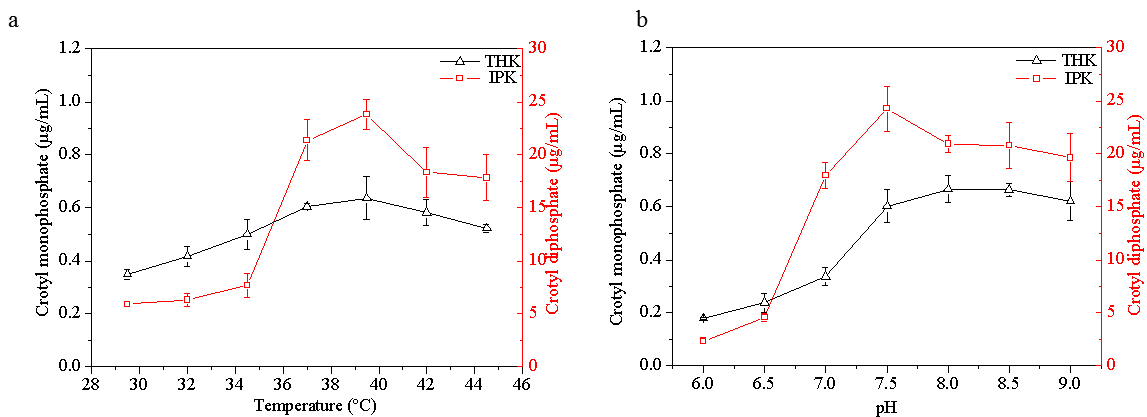
**

**Fig. S3** Determining the optimal temperature and pH for THK and IPK.

**a** The optimal temperature of THK and IPK. **b** The optimal pH of THK and IPK . Data represent mean and standard deviations calculated from three biological replicates.


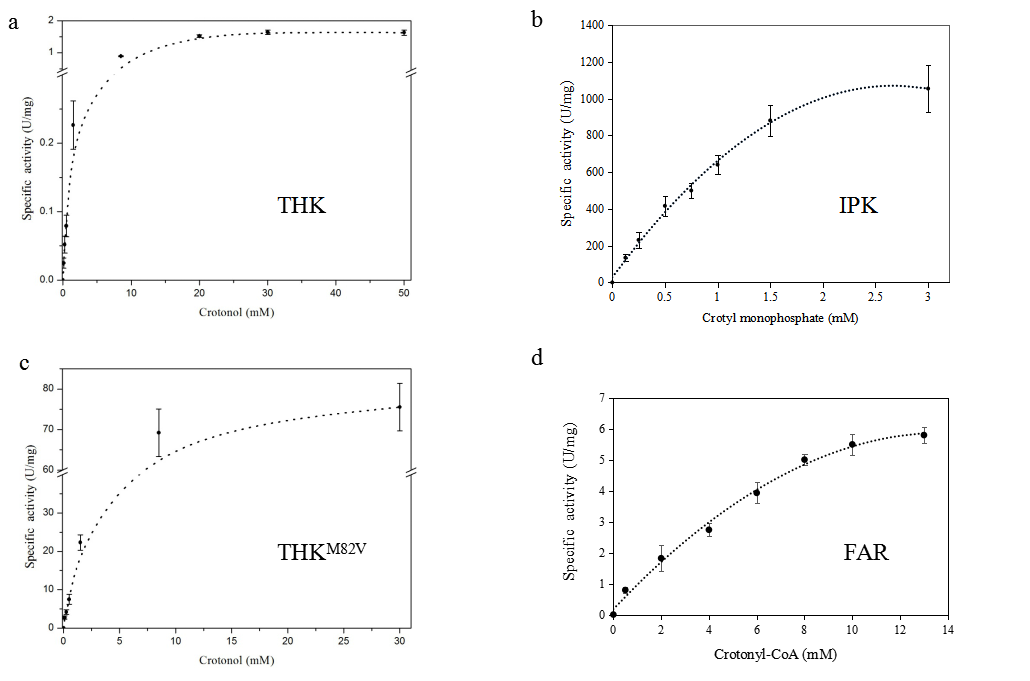


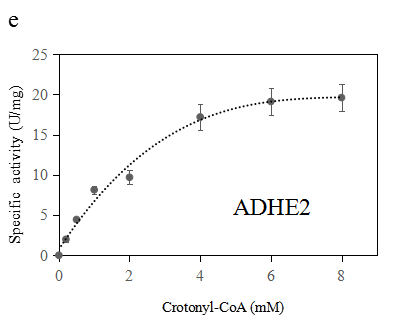


**Fig. S4** Enzymatic kinetics of THK, THK^M82V^, IPK, FAR and ADHE2.

**a** Wild-type THK towards crotonol. **b** IPK towards crotyl monophosphate. **c** THK^M82V^ towards crotonol. **d** FAR towards crotonyl-CoA. **e** ADHE2 towards crotonyl-CoA Data represent mean and standard deviations calculated from three biological replicates.

a b


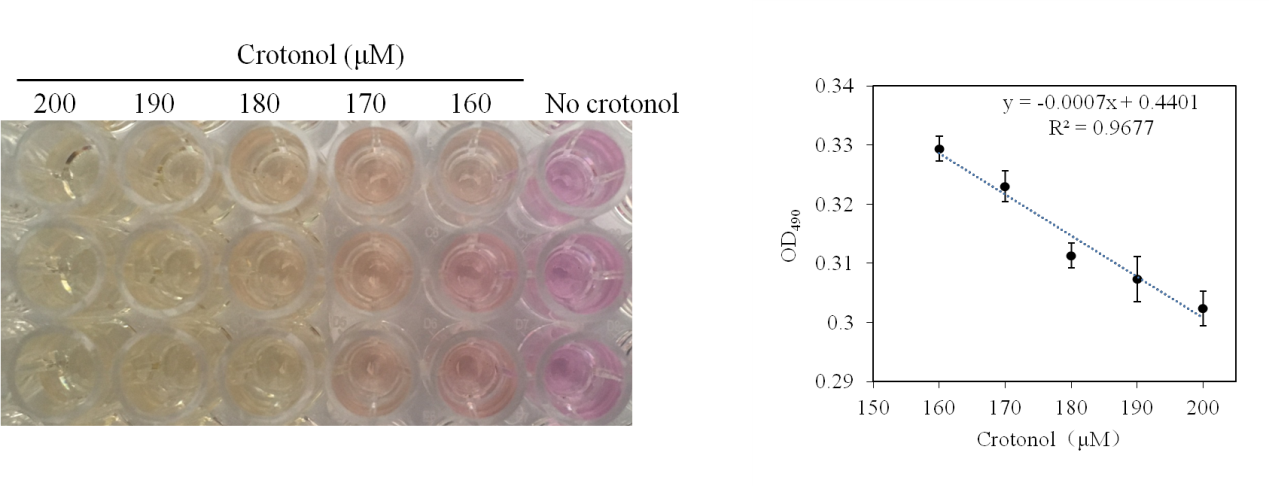


**Fig. S5. Development of a high throughput screening method.**

Wild-type *M. extorquens* AM1 was grown on succinate to mid-exponential phase (OD_600_=0.60), then crotonol from 160 to 200 mM was added into the culture medium. The supernatants were then transferred into 96-well plate and potassium permanganate was added at a final concentration of 200 μM.

**a** Color reaction between crotonol and potassium permanganate for 3 min.

**b** The linear correlation between crotonol concentration and OD_490_ value. Data show the mean with error bars indicating standard deviation calculated from three independent biological replicates.


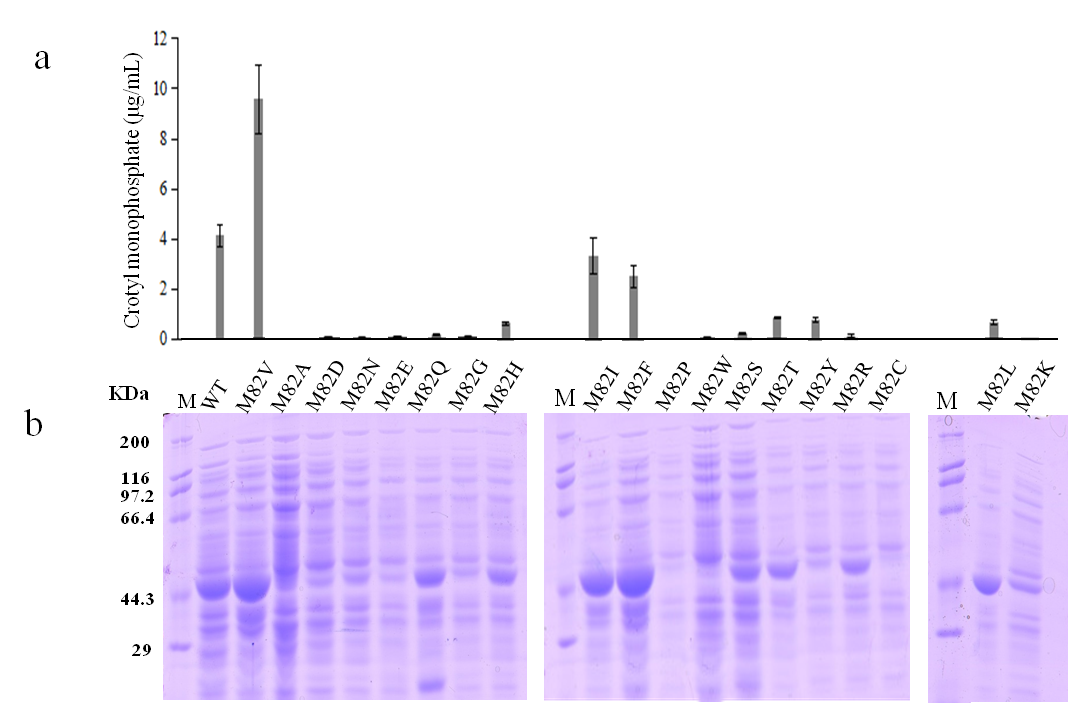


**Fig. S6. The effect of targeted mutation on THK activity.**

**a** Activity for mutants of the 82^th^ amino acid of THK. A crude enzymatic assay detects the production of crotyl monophosphate by LC-MS at 1 h.

**b** Crude proteins extracted from *E. coli* are analyzed by SDS-PAGE gel.

Data show the mean with error bars indicating standard deviation calculated from three independent biological replicates.

**
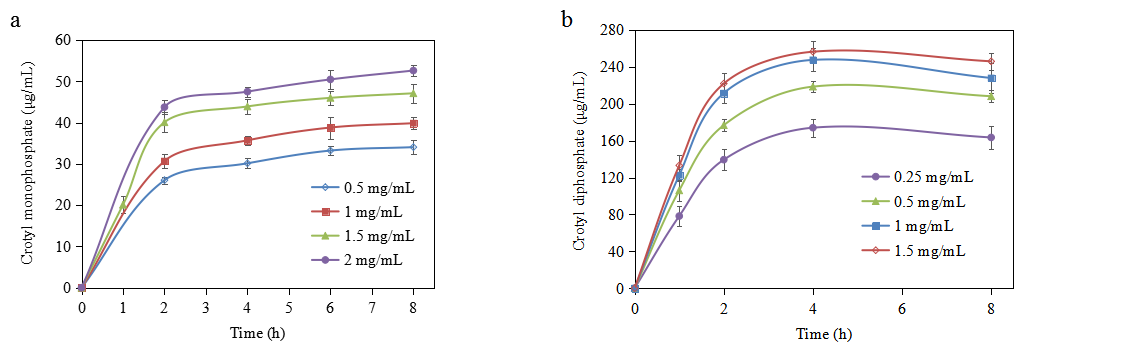
**

**Fig. S7** The effect of kinase concentrations on the production of crotyl monophosphate and crotyl diphosphate *in vitro.*

**a** Time and concentration curves of crotyl monophosphate with 10 mM (720 μg/mL) crotonol as substrate and catalyzed by loading different concentrations of THK^M82V^. **b** Time and concentration curves of crotyl diphosphate with 4 mM (608 μg/mL) crotyl monophosphate as substrate and catalyzed by adding different concentrations of IPK. Data represent mean and standard deviations calculated from three biological replicates
